# Biologic Therapies across Nasal Polyp Subtypes

**DOI:** 10.3390/jpm14040432

**Published:** 2024-04-19

**Authors:** Kody G. Bolk, Sarah K. Wise

**Affiliations:** Department of Otolaryngology—Head and Neck Surgery, Emory University Hospital Midtown, Emory University, Atlanta, GA 30308, USA

**Keywords:** nasal polyps, chronic sinusitis, biologic, allergic fungal rhinosinusitis, central compartment atopic disease, aspirin-exacerbated respiratory disease

## Abstract

Chronic rhinosinusitis with nasal polyposis is a common inflammatory condition, with subtypes like aspirin-exacerbated respiratory disease, allergic fungal rhinosinusitis, and central compartment atopic disease sharing a common type 2 inflammatory pathway. Respiratory biologic therapies have been developed that target type 2 inflammation. In this article, we discuss the use of respiratory biologic therapies for nasal polyposis in general, as well as within the various subtypes of nasal polyps. Further, we discuss future roles of novel biologic therapies targeting type 2 inflammation in nasal polyposis.

## 1. Introduction

Chronic rhinosinusitis (CRS) is relatively common condition, affecting 4.5 to 12% of individuals in the United States and European countries [1,2,3,4]. It is an inflammatory condition of the sinonasal cavity characterized by symptoms of nasal obstruction, nasal discharge, hyposmia, or facial pain/pressure for durations greater than 12 weeks. Objective findings are necessary for diagnosis and include edema and/or mucopurulence on endoscopy or computed tomography (CT) findings suggestive of chronic inflammation [5,6].

CRS has classically been divided into phenotypic subgroups based on the presence or absence of nasal polyposis. Further classification of CRS relies on associated clinical characteristics and endotypes. Endotypes, or inflammatory profiles, have been primarily classified in three different groups, with some overlap. These include neutrophil-driven inflammation, mediated by T-helper (Th)1 inflammation from interleukin (IL)-12 and interferon gamma. Next is Th2-mediated inflammation predominantly driven by IL-4, IL-5, and IL-13. Immunoglobulin (Ig) E also influences type 2 inflammation. Last is Th17/22 mediated by IL-17 and IL-22 promoting neutrophil and macrophage inflammation [5,7,8,9,10]. 

Additional disease categorization for chronic rhinosinusitis with nasal polyposis (CRSwNP) has been described as well. This includes subtypes such as aspirin-exacerbated respiratory disease (AERD), also known as non-steroidal anti-inflammatory drug-exacerbated respiratory disease (NERD), allergic fungal rhinosinusitis (AFRS), central compartment atopic disease (CCAD), and those not otherwise falling into these categories or not otherwise specified (CRSwNP NOS). The AERD/NERD, AFRS, CCAD, and CRSwNP NOS subgroups cause disease mainly through a type 2 inflammatory response. It is important to understand this mechanism of inflammation in order to understand biologic therapies for treatment. Patients with other systemic disease entities may also present with nasal polyps, such as cystic fibrosis (CF), although this is not primarily mediated through type 2 inflammation and would not be anticipated to respond well to respiratory biologics in the Th2 inflammatory space, so proper diagnosis is critical.

Respiratory epithelial cells are important as these can lead to an inflammatory cascade of type 2 inflammation. Barrier dysfunction is prompted by insults such as stress or environmental triggers. This triggers alarmins like IL-25, IL-33, and thymic stromal lymphopoietin (TSLP) and perpetuates the type 2 inflammatory cycle [11]. Type 2 inflammatory cells are stimulated to produce IL-5, which drives eosinophilia [12]. IL-4 and IL-13 are also released, and both signal through the IL-4 receptor alpha, stimulating mucus production, goblet cell hyperplasia, tissue fibrosis, as well as local IgE production. IgE provokes mast cell degranulation and release of inflammatory mediators inducing mucosal edema and obstruction [12,13]. IL-5 and its receptor (IL-5R) signal eosinophil activation and survival, but the receptors may also be found on other local polyp cells like plasma cells. This suggests there may be other drivers of inflammation outside of IgE production, as demonstrated by the observation that when IgE was depleted from nasal tissue, polyps did not regress [14,15].

Based on the understanding of the activation and perpetuation of the type 2 immune pathway, biologic therapies have been utilized for disruption of the cascade and treatment of disease inflammation. Biologic therapies targeting type 2 inflammation have been used to treat various diseases including asthma, eosinophilic granulomatosis with polyangiitis, hypereosinophilic syndrome, atopic dermatitis, prurigo nodularis, eosinophilic esophagitis, and nasal polyps, among others [16,17,18,19,20]. Dupilumab is a monoclonal antibody that inhibits IL-4 receptor alpha, which is utilized in the IL-4 and IL-13 signaling pathways integral to Th2 inflammation [6]. Omalizumab is a monoclonal antibody against IgE that inhibits binding to IgE receptors on mast cells and basophils and decreases the release of mediators for allergic inflammation [6]. Mepolizumab and reslizumab are monoclonal antibodies that target IL-5 directly. Benralizumab is a monoclonal antibody that binds the IL-5R alpha subunit on eosinophils. This inhibits IL-5 activity mediating chemotaxis, differentiation, activation, and survival of eosinophils (anti-IL-5) and blocks effects on mediators like mast cells and B cells (anti-IL-5R alpha) [6]. 

Indications for biologics for CRSwNP based on the EPOS 2020 document have been outlined for patients with bilateral polyps who have previously undergone endoscopic sinus surgery or are unfit to undergo surgery. Three of the following criteria are required for those wishing to initiate biologic therapy with a biologic that targets type 2 inflammation. These include:

1. Evidence of type 2 inflammation such as tissue eosinophils ≥10/hpf, blood eosinophils ≥250 cells per microliter, or total IgE ≥ 100 IU/mL
2. Need for systemic corticosteroids (SCS) including ≥2 courses per year or long term (>3 months)
3. Significantly impaired quality of life (SNOT-22 ≥ 40)
4. Significantly impaired sense of smell (anosmic on smell test)
5. Diagnosis of comorbid asthma (requiring inhaled corticosteroids) [21].

EPOS guidelines for indication of biologic therapy for CRSwNP underwent an update in 2023. A change in the blood eosinophil cutoff of ≥150 cells per microliter was recommended [22].

In the sections that follow, we discuss the usage and efficacy of currently available biologic therapy for various subtypes of nasal polyps.

## 2. Biologic Use across Nasal Polyp Subtypes

### 2.1. Chronic Rhinosinusitis with Nasal Polyposis

CRSwNP NOS encompasses patients with nasal polyposis not categorized into other specific subtypes. In this discussion, we review the studies conducted to investigate the efficacy of individual biologic therapies for CRSwNP NOS.

Investigation into treatment for CRSwNP with dupilumab has been undertaken in two large randomized, double-blind, placebo-controlled, parallel-group studies named SINUS-24 and SINUS-52 [4]. As previously noted, dupilumab interferes with signaling of IL-13 and IL-4, which contribute to downstream effects of type 2 inflammation. Patients in SINUS-24 and SINUS-52 studies were 18 or older who had bilateral nasal polyps with scores greater than 5, along with persistent disease despite intranasal corticosteroids (INCS) or systemic corticosteroids (SCS) in the last 2 years or prior sinonasal surgery. Patients also were screened for symptoms of nasal congestion or obstruction as well as loss of smell or nasal discharge. Treatment period with dupilumab and INCS was for 24 weeks in SINUS-24 and 52 weeks in SINUS-52 with follow-up periods of 24 weeks and 12 weeks, respectively. In total, 724 patients were included between the two trials. Sixty-four to eighty-one percent of patients had previously received SCS, and 58–74% had received prior sinonasal surgery [4].

In each trial, the endoscopic nasal polyp score (NPS) was significantly improved in the dupilumab-treated group compared to the placebo group. Forty-six percent of patients in each study treated with dupilumab noted at least a two-point improvement in NPS. Nasal congestion score (NCS) significantly improved, starting early (4–8 weeks) and lasting through each trial. These metrics also improved in the cohorts with comorbid asthma, NERD, and those with prior surgery. Further, SNOT-22, UPSIT, and Lund-Mackay scores all improved in the dupilumab-treated group compared to the placebo group. This improvement was greater for those patients treated with every 2-week dosing compared to every 4-week dosing in SINUS-52. In the SINUS-24 cohort, beneficial treatment effects were noted to diminish in the follow-up periods after the drug was discontinued, but those treated with dupilumab for 52 weeks noted sustained improvement throughout. Based on this finding, it has been advocated to continue treatment with dupilumab long term to suppress type 2 inflammatory effects. Overall, dupilumab was very well tolerated throughout [4]. It is important to note that the patients in this study treated with dupilumab noted improvements in objective and patient report outcomes measures. These outcomes have not always correlated, and this may reflect the broad ability for dupilumab to target type 2 inflammation [23]. Analysis further noted that up to 95% of patients displayed characteristics of type 2 inflammation. Varying definitions of type 2 inflammation included eosinophils ≥150 cells/μL or IgE ≥ 100 IU/mL with a coexisting T2 condition; eosinophils ≥150 cells/μL or IgE ≥ 100 IU/mL; eosinophils ≥150 cells/μL; eosinophils ≥250 cells/μL or IgE ≥ 100 IU/mL; coexisting asthma or eosinophils ≥300 cells/μL; presence of a coexisting T2 condition [24]. However, patients with severe CRSwNP still benefited from dupilumab treatment, regardless of degree of eosinophilic status [25]. This treatment can be considered for those with CRSwNP not otherwise specified that have recalcitrant disease despite thorough surgery and maximal medical therapy. FDA approval for dupilumab use in nasal polyps was granted in 2019.

Blockage of type 2 inflammation through inhibition of IgE with omalizumab has been investigated in nasal polyps as well. In a study by Gavaert et al., 24 patients with asthma and CRSwNP underwent a randomized, double-blind trial of omalizumab versus placebo. At 16 weeks, there was a significant improvement in NPS, as well as nasal and asthma symptom scores, in those treated with omalizumab compared to placebo [26]. This is hypothesized to be due to effects of anti-IgE treatment on local tissue IgE in nasal polyps, which is independent of total IgE. This local polyclonal IgE effect on tissue may induce the inflammation in the airway in asthma and CRS patients, even in allergic and nonallergic patients [26,27]. 

Further investigation into type 2 inflammatory blockade through inhibition of IgE with omalizumab for treatment of CRSwNP was conducted in the POLYP 1 and POLYP 2 trials [28]. These were randomized, multicenter, double-blind, placebo-controlled trials investigating omalizumab in patients with CRSwNP despite daily INCS treatment. Patients were required to have at least four weeks INCS treatment, total NPS of 5, NCS greater than 2 and SNOT-22 score of 20 or higher. Patients were treated with omalizumab plus intranasal mometasone versus placebo and intranasal mometasone [28]. One-hundred thirty-three patients completed the POLYP 1 trial with 69 patients in the omalizumab arm, while 121 patients completed the POLYP 2 trial with 58 patients in the omalizumab arm. Twelve to twenty-nine percent of patients had prior systemic corticosteroid treatment, and 59.6% underwent prior sinonasal surgery. At 24 weeks, mean NPS decreased by 1.08 in the omalizumab group compared to an increase of 0.06 in the placebo group, which met statistical significance. Daily NCS also improved significantly for the omalizumab-treated cohort. Significant improvements were also seen in omalizumab-treated patients in the SNOT-22 score, UPSIT score, total nasal symptom score, and individual nasal symptoms like smell, postnasal drip, and runny nose. Reduced need for surgery was seen in the omalizumab group at week 24. Omalizumab was well tolerated throughout. Based on these results, omalizumab may be considered for patients with refractory CRSwNP [28]. Omalizumab is FDA approved for use of nasal polyps as of 2020.

Mepolizumab was investigated for CRSwNP patients in the SYNAPSE trial, a randomized, double-blind, placebo-controlled, parallel-group, phase-3 trial done at 93 centers. In this study, 414 patients were randomly assigned with 407 included in the intention-to-treat population. Two-hundred and six patients received mepolizumab and two-hundred and one received a placebo for 52 weeks. Patients all had prior sinus surgery and were given mepolizumab over the course of a year. NPS and nasal obstruction scores were significantly decreased from baseline in the treatment arm. Patients in the mepolizumab group had a significantly larger portion of patients with one point or higher improvement in NPS (50%) compared to 28% for placebo. SNOT-22 scores and need for surgery were also decreased in the mepolizumab group. Blood eosinophil counts were significantly decreased in the mepolizumab group with 81% reduction at week 4, and this was maintained through week 52. Adverse effects were similar between the two groups, with common events being nasopharyngitis, sinusitis, headache, and epistaxis [29]. A follow-up study at 24 weeks after discontinuation of mepolizumab in this cohort noted sustained clinical improvements with decreased NPS and need for surgery compared to placebo. Blood eosinophil counts returned to baseline. Patients still noted improvement in symptom scores like nasal obstruction and SNOT-22, but the magnitude of improvement waned in the follow-up period (Table 1) [30]. Mepolizumab gained FDA approval for treatment of nasal polyps in 2021. An additional randomized controlled trial of mepolizumab in CRSwNP was conducted with 105 patients randomly assigned treatment with mepolizumab or placebo. After 25 weeks, the mepolizumab-treated group noted improvements in VAS, endoscopic polyp scores, and SNOT-22 scores compared to the placebo group. There was also a reduction in the proportion of patients requiring surgery in the mepolizumab group compared to the placebo group [31].

Reslizumab is another inhibitor of IL-5 that has been investigated for treatment of CRSwNP [32]. Twenty-four patients were randomly assigned to treatment with a single dose of reslizumab at two different concentrations versus placebo. After regular follow-up intervals up to 36 weeks, there was no significant difference in symptom scores or nasal peak inspiratory flow. Individual NPS improved in half of the patients treated with reslizumab, and these patients were deemed responders. Responders were noted to have a decrease in nasal IL-5 compared to non-responders; however, both groups showed a decrease in blood eosinophil counts for up to 8 weeks. Currently, reslizumab is not FDA approved for use in nasal polyps [32].

Differences are noted between the biologic studies on nasal polyposis when comparing inclusion criteria, especially in the criterion of prior sinus surgery for patients. In the SINUS-24 and SINUS-52 trials, patients were included with prior surgery or persistent disease despite INCS or SCS in the last two years [4]. In the POLYP1 and POLYP2 trials, there was no specified criteria for prior surgery for included patients [28]. Further, in the SYNAPSE trial, all patients had prior surgery [29]. While most patients would be recommended for sinus surgery in the standard of care for nasal polyposis, there still remains the lack of consensus of extent of sinus surgery. This may vary from complete, extensive surgery to address all paranasal sinuses affected versus polypectomy or more limited surgery. Future studies are needed to aid in standardization of extent of surgery for better comparisons of biologics and effects on CRSwNP.

Aside from the described trials leading to FDA approval for dupilumab, mepolizumab, and omalizumab for nasal polyps, several additional studies have been published, including real life experiences, evaluating efficacy [33,34,35,36,37,38,39,40,41,42,43,44,45,46] and safety [47,48,49,50,51,52] of biologics use in CRSwNP. Long term outcomes are becoming available as well [44,53,54,55,56]. One significant gap that remains, however, is understanding the need for biologic therapy continued indefinitely.

### 2.2. Aspirin-Exacerbated Respiratory Disease (AERD/NSAID-ERD)

AERD is a subtype of CRSwNP with criteria of asthma, nasal polyposis, and sensitivity to aspirin or NSAIDs. Some prefer the term NERD to AERD, although we use AERD here. CT imaging typically shows pansinus opacification, and the gold standard for diagnosis is aspirin challenge. These patients have increased production of leukotriene E4, which leads to recruitment of eosinophils and type 2 inflammation [57]. This is a non-IgE-mediated sensitivity to COX-1 inhibitors [58,59,60,61]. AERD is not typically classified as an atopic disorder, but recent evidence suggests nasal polyp regrowth is related to nasal polyp tissue IgE levels. IgE may cause continued respiratory inflammation and mast cell activation [13]. Further, local activation of IL-5 and IL-5R from other inflammatory cells like plasma cells may lead to increased inflammation in this cohort of patients [13].

Management of AERD includes surgical and medical therapies. Endoscopic sinus surgery (ESS) is typically advocated, and often multiple surgeries are performed for recalcitrant disease [61]. Medical therapy includes avoidance of COX-1 inhibitors, aspirin desensitization with daily aspirin therapy, INCS, nasal saline irrigations, and consideration of leukotriene modifying agents [57,61,62,63].

Biologic therapies can be considered for the treatment of AERD as well. Dupilumab effects on AERD disease control have been documented as a result of the dupilumab clinical trial for CRSwNP patients (NCT01920893). In this cohort of 60 patients, 19 self-reported comorbid AERD with sensitivity to aspirin or NSAIDs. These AERD patients had increased smell loss, high Lund-MacKay scores, and decreased mean FEV1 compared to others in the study. NPS was similar in patients with and without AERD. AERD patients had a significant decrease in NPS from baseline to 16 weeks. There was also a decrease in NPS in this timeframe for non-AERD patients, but this did not reach significance. Both AERD and non-AERD patients treated with dupilumab had decreased Lund-MacKay score, improved SNOT-22 score, improved asthma scores, and improved lung function. Overall, the patients with AERD had worse disease at baseline compared to those that were aspirin tolerant, yet had significant improvements in CRS and asthma scores [64]. Based on the SINUS-24 and SINUS-52 study cohorts, 28.2% of patients were noted to have NERD. Those treated with dupilumab noted significantly greater improvements in nasal congestion scores, SNOT-22 scores, total symptom scores, and peak nasal inspiratory flow [65].

Anti-IgE treatment with omalizumab has also been evaluated in AERD patients. In a study of 21 patients treated for AERD with omalizumab for one year, 85% were noted to be responders and experience significant decreases in nasal symptom scores, did not require sinus surgeries, and had reductions in corticosteroid usage, asthma scores, and lower airway exacerbations [66].

Anti-IL-5 and anti-IL-5R biologics may show efficacy in AERD patients for disease and symptom control due to reduction in tissue eosinophilia, which has been beneficial for respiratory symptoms in AERD. As mentioned, reduction of eosinophils alone does not improve nasal polyp burden. However, these biologics may offer other effects like playing a role in reduction of epithelial mast cell leukotriene E4 production [13]. Mepolizumab was investigated for CRSwNP patients in the SYNAPSE trial, and within this trial, 45 patients in the treatment group had comorbid AERD compared to 63 patients with AERD in the placebo group. NPS and nasal obstruction scores were significantly decreased from baseline in the treatment arm. SNOT-22 scores and need for surgery were also decreased in the mepolizumab group. 

AERD patients may benefit from concurrent aspirin therapy after desensitization as well as biologic therapy. In a study surveying 98 patients with AERD, 53% reported use of respiratory biologic including mepolizumab, omalizumab, dupilumab, benralizumab, or reslizumab, and 86% reported undergoing aspirin therapy. Twenty-four patients noted concurrent use of aspirin and biologic therapy. Typically, this was completed for older patients or those with lack of symptom control on aspirin therapy alone, prompting initiation of biologic therapy [67].

### 2.3. Allergic Fungal Rhinosinusitis

In 1994, Bent and Kuhn published the major criteria for AFRS including nasal polyps, fungal elements present on staining, eosinophilic mucin without fungal invasion, type 1 hypersensitivity to fungi, and characteristic radiographic findings (unilaterally or asymmetric findings, bony remodeling, and heterogenous densities within the sinuses) [68]. Minor criteria include bone erosion, Charcot–Leyden crystals, unilateral disease, peripheral eosinophilia, positive fungal culture, and lack of an immunodeficient state [68,69]. Thick brown/yellow mucin as well as nasal polyposis is noted on endoscopy [5]. These patients are typically younger at presentation and found in regions with high humidity and warmer temperatures [70,71].

Management of AFRS typically includes ESS to remove polyps and mucin and allow corticosteroid irrigations to access the sinus spaces. Allergen immunotherapy may also be considered [5,72,73,74,75,76]. There have been several studies that report use of biologic therapy for AFRS, although AFRS patients were excluded from the large clinical trials of biologics for nasal polyposis that led to FDA approval of many of the therapies already discussed. Dupilumab use has been reported in AFRS, and efficacy is thought to be due to blockage of IL-4 and IL-13 cascade, which is common to type 2 inflammation and AFRS. A case report by Alotaibi et al. noted success when treating a patient with AFRS that had failed maximal medical therapy and four prior endoscopic sinus surgeries. The authors noted a significant decline in eosinophil count, improvement in symptom scores, and notable improvement on endoscopy [77]. Further, a retrospective case series investigated dupilumab effects on four patients with AFRS and comorbid asthma, for which the biologic was initiated. These patients had failed prior sinus surgeries, oral corticosteroids, topical corticosteroids, and nasal irrigations. There was notable improvement in nasal symptom scores, need for steroids for asthma control, pulmonary function, and CT scores of the paranasal sinuses after dupilimab [78].

Mepolizumab has also been utilized for patients with AFRS and comorbid asthma. In one retrospective chart review, 27 patients with AFRS and asthma were treated with mepolizumab injections monthly and follow up was every 6–8 weeks for three visits. There was significant improvement in endoscopic scores, especially for those with greater polyposis burden. Decreases in SNOT-22 scores and eosinophil counts were also noted [79].

AFRS shares similarities with type 2 inflammatory endotypes of CRSwNP. However, this disease process in particular is known for significant elevation of serum IgE [12]. Therefore, omalizumab has been investigated for treatment of AFRS. A two-armed, single blinded, prospective randomized trial was completed for patients with AFRS with 10 patients in one arm treated with omalizumab and 10 patients in the other arm treated with INCS 2 weeks postoperatively from sinus surgery [80]. At 24 weeks, there were significantly lower SNOT-20 scores and total nasal symptom scores for the omalizumab group. There was greater reduction in the total IgE in the omalizumab treatment arm, but this did not reach significance. There were no differences in postoperative endoscopic scores or complication rates [80]. Additionally, a retrospective case series of patients with AFRS and refractory asthma was investigated for effects with omalizumab. Seven patients had failed prior surgery, topical and oral corticosteroids, and antifungals. Patients were treated with omalizumab every 2 weeks for a mean follow up of 9.7 months. There was a 31% improvement in mean SNOT-22 scores, an improvement of 61% in endoscopic scores, and no patients required further surgery. There was no significant difference in pre-omalizumab and post-omalizumab IgE levels [81].

Currently, there is limited data on efficacy of biologics for AFRS. To address this deficiency, an ongoing randomized controlled trial is evaluating the role of dupilumab for recurrent symptomatic nasal polyps in patients with AFRS who have undergone at least one sinus surgery (NCT04684524) and another as adjuvant therapy after sinus surgery (NCT05545072) [78]. NCT05545072 is investigating dupilumab versus placebo in the postoperative setting. Patients will undergo randomization into the two arms and continue on standard of care therapy with INCS and either dupilumab or placebo [82]. NCT04684524 is a randomized trial of dupilumab versus placebo for AFRS patients. The outcomes include opacification on imaging, symptom scores, efficacy, NPS, smell outcomes, among others [83].

### 2.4. Central Compartment Atopic Disease (CCAD)

Central compartment atopic disease is an additional subgroup of CRSwNP. CCAD is characterized by central nasal cavity polypoid changes along the posterior/superior nasal septum, middle turbinates, and/or superior turbinates [5,84]. Patients present with symptoms of CRS as well as nasal itching and sneezing, and there is a strong association with allergy in early reported series [7,8,85]. Common CT findings include soft tissue thickening in the central nasal cavity with sparing of the sinuses laterally. Later in the disease, centrally-located polyps may cause lateralization of the turbinates and subsequent sinus involvement [5,86].

ESS is employed to remove polypoid changes within the central compartment. The paranasal sinuses may be opened if involved as well. Other medical management includes corticosteroid irrigations, as well as possibly oral and topical anti-histamines or allergen immunotherapy, but further study is needed [7,86,87].

Thus far, there does not appear to be a regular role for biologic therapy for CCAD. Compared to other subgroups of CRSwNP, these patients typically require fewer surgeries due to overall decreased disease burden and lower rate of polyp recurrence [87]. Further, when comparing inflammatory markers to AFRS and AERD, CCAD patients had significantly lower type 2 cytokines (IL-5 and IL-13) and lower tissue eosinophil counts [88]. Due to the unique characteristics of CCAD patients with a lower inflammatory burden, disease can typically be managed without the use of biologics. Further investigation will be necessary to determine future roles.

## 3. Future Directions

Hellings et al. conducted an overview of studies that evaluated biologic therapy for CRSwNP. They noted difficulty in direct comparisons between biologics since inclusion criteria, outcomes, and time points of analysis varied. They suggested that registries, comparative trials, or endotype-driven studies would be beneficial to further compare biologic therapies effectively [89]. Additionally, Papacharalampous et al. conducted a systematic review of 37 studies comparing omalizumab, mepolizumab, and dupilumab in treatment of CRSwNP [90]. They noted a moderate advantage in usage of dupilumab over omalizumab and mepolizumab in terms of clinical findings and symptom scores as well as need for rescue surgery. However, this is overall of relatively low evidence as head-to-head trials, large comparative multicenter trials, and durability of effect over follow-up periods remain to be studied [90]. Currently, clinical trial NCT04998604 is investigating dupilumab versus omalizumab versus placebo in patients with refractory CRSwNP in a randomized, double-blind trial with the primary outcomes of smell change and NPS [91]. Included patients are those 18 years or older with severe CRSwNP and comorbid asthma. Patients must have a NPS of 5 or greater, symptoms of nasal congestion and loss of smell, and physician-diagnosed asthma greater than 12 months. Patients having had sinonasal surgery within the last six months are excluded [92].

Other novel biologic agents are currently under investigation for use in CRSwNP. First, tezepelumab is a human monoclonal antibody that inhibits the thymic stromal lymphopoietin receptor (TSLP). TSLP is activated in response to inflammatory or chemical stimuli of the epithelium. TSLP then stimulates innate lymphocytes, mast cells, basophils, and Th2 cytokine release leading to type 2 inflammation [93]. Tezepelumab was initially approved for treatment of severe asthma in adults. It was shown to decrease exacerbations and improve lung function, asthma control, and quality of life compared to placebo [94]. Clinical trial NCT04851964 is investigating efficacy of tezepelumab on patients with nasal polyps. Primary outcomes are change in nasal polyp score and change in NCS from baseline [95].

Depemokimab is a long-acting human monoclonal antibody against IL-5. This is currently under clinical trial investigation compared to the placebo arm for efficacy in CRSwNP with primary outcomes of endoscopic NPS and nasal obstruction score compared to the baseline (NCT05281523) [96].

At this time, respiratory biologics that mediate changes in type 2 inflammation have shown efficacy in subtypes of CRSwNP including CRSwNP NOS, AERD, and AFRS, although the types of studies and outcomes vary. Future trials may show efficacy for newer therapies, and comparisons between biologics may lead to greater insights in choosing the appropriate therapy for individual patients.

## Figures and Tables

**Table 1:** Comparison of large-scale randomized controlled trials of respiratory biologics targeting type 2 inflammation in nasal polyps.

Randomized Controlled Trial	Biologic Agent	Target	Eligibility Criteria	Outcomes	Side Effects
SINUS-24 SINUS-52 [4]	Dupilumab	IL-4 receptor alpha	Bilateral nasal polyposis, NPS greater than 5, failed topical and systemic corticosteroids in last 2 years or prior sinonasal surgery	Improved nasal polyp, UPSIT, SNOT-22, and Lund-Mackay scores in treatment arm	Nasopharyngitis, worsening of nasal polyps or asthma, headache, epistaxis, injection site erythema
POLYP 1 POLYP 2 [28]	Omalizumab	IgE	Failure of 4 weeks of INCS, total NPS of at least 5, NCS greater than 2, SNOT-22 score of 20 or greater	Decreased NPS; significant improvements in nasal congestion, SNOT-22, UPSIT, and total nasal symptom scores; reduced need for surgery at week 24 in treatment arm	Headache, injection site reaction, arthralgia, dizziness, and upper abdominal pain
SYNAPSE [29]	Mepolizumab	IL-5	Refractory nasal polyps, VAS nasal obstruction score greater than 5, eligible for repeat nasal surgery despite standard of care; at least one prior sinonasal surgery, CRS symptoms despite INCS	Significant reduction in nasal polyp, nasal obstruction, and SNOT-22 scores; decreased need for surgery and blood eosinophil counts in the treatment arm	Nasopharyngitis, sinusitis, headache, epistaxis

## Data Availability

No new data were created or analyzed in this study. Data sharing is not applicable to this article.
